# Spanish validation of female condom attitude scale and female condom use in Colombian young women

**DOI:** 10.1186/s12905-019-0825-z

**Published:** 2019-10-28

**Authors:** Vallejo-Medina Pablo, Ramírez Carlos Eduardo, Saavedra-Roa Diego Alejandro, Gómez-Lugo Mayra, Pérez-Durán Claudia

**Affiliations:** 1 SexLab KL, Fundación Universitaria Konrad Lorenz, Dir: Cra. 9 Bis #62-43, Bogotá, Colombia; 2 School of psychology, Fundación Universitaria Konrad Lorenz, Dir: Cra. 9 Bis #62-43, Bogotá, Colombia

**Keywords:** Female condom, Perception, Psychometric validation, Attitude

## Abstract

**Background:**

Infection by HIV and other STIs and unplanned pregnancies are among the most serious problems associated with sexuality. Male and female condoms are the only dual-purpose devices to control both unplanned pregnancies and STIs, and studying people’s attitudes toward the use of these devices are excellent ways to predict their use. Therefore, the purpose of the present study was to adapt and validate the Female Condom Attitude Scale for Spanish language and to evaluate the use of female condoms in Colombian population.

**Methods:**

For that purpose, a total of 387 Colombian women aged 23.68 years in average were asked to respond to the Female Condom Attitude Scale, the Sexual Opinion Survey, and the UCLA Multidimensional Condom Attitudes Scale.

**Results:**

The use of female condom in Colombia is very low; only 5.10% of the surveyed women had used it at least once. On the other hand, results revealed a five-factor dimensionality (Sexual pleasure enhancement, Inconvenience, Improved prophylaxis, Sexual pleasure inhibition, and Insertion reluctance) with alphas between .81 and .96. The scale also showed adequate psychometric properties and criterion validity. No relationship was found between attitudes toward female condom and attitudes toward male condom.

**Conclusions:**

The Spanish adaptation of the Female Condom Attitude Scale was found to be reliable and valid in a sample of young women.

## Background

Infection by HIV is one of the most serious sexual health problems. The World Health Organization [1] reported a total of 36,700,000 people infected with HIV in the world; 2.1 million of these people became infected in 2015. Approximately 2300 Colombian citizens have died due to AIDS [2], which has resulted in 23,000 orphaned children, and the prevalence of HIV among people from 14 to 49 years of age is 0.5% (150.000). Besides HIV, the recent increase of other Sexually Transmitted Infections (STIs) should be taken into account. Worldwide, the quality of life of many people has been affected by STIs, especially in low- and medium-income countries [3, 4]. An annual average of 98,423 cases of STIs were reported in Colombia in 2015, 23% of which were estimated to be of the ulcerative type [5]. On the other hand, Colombia is one of the Latin American countries reporting the highest numbers of teenage pregnancies [6]. It is estimated that 29.2% of women who gave birth were unprepared, and that 21.3% of pregnancies were undesired. Consequently, 50.5% of natality in Colombia could be considered as unplanned [6].

Male and female condoms are the only dual control device: they reduce the transmission of HIV and other STIs and they also prevent unplanned pregnancies [7]. Therefore, the use of condoms must be promoted in order to prevent STIs and unplanned pregnancies [1, 2, 4, 7]. A recent study in Colombia showed that only 22% of adolescents (14–19 years old) were using condoms consistently [8]; this percentage differs from Colombian data for previous years (30 to 42%) [9, 10] and from data obtained in other countries (40 to 54%) [11, 12]. These figures highlight the need to look for alternatives to the male condom in order to counteract its decreasing use. The prevalence of the use of the female condom in Colombia is unknown, but it was found to be an important component to be used a sexual health promotion program: “*Interventions should not only teach the male one (condom), but also the female one because it is also important*” (Marta, Bogotá), or “*Well, I have heard about female condoms, but I thought that it was an urban myth, I didn’t know they actually existed ( …*)” (Joseph, Barranquilla) [13]. These observations are also indicators of the importance of promoting the female condom.

Condom use can be predicted on the basis of psychosocial variables. Some of the most important are behavioral intentions, communication about condoms, condom use self-efficacy, and knowledge or attitudes toward condoms [14]. Attitudes are the result of the inclination or tendency to produce a favorable or unfavorable conscious response toward, in this case, an object; they reflect an evaluation of the object made by an individual in connection with dimensions such as good or bad, beneficial or harmful, pleasant or unpleasant, agreeable or disagreeable, etc. [15]. Attitudes are essential elements in one of the main STD and unintended pregnancy prevention theories like Planned Action Theory [15] and is related with other theories like: Theory of Behavioral Change and Information-Motivation-Behavioral Skills Model-Based theory [16, 17]. For the Planned Action Theory, intention is the best predictor of behavior and is determined by attitudes, normative beliefs towards those behaviors and the perception of control. The attitudinal component is defined as the set of beliefs about the value of behavior and its consequences. The attitude towards condom use is more favorable when the negative consequences of not using a condom (contracting an STI or unwanted pregnancy) and the benefits of its use (use of the condom as an exciting element) are assessed [18].

The evaluation of attitudes toward condoms, both male and female, is extremely important because attitudes and perceptions toward one or the other are not necessarily the same [10, 19]. Therefore, attitudes toward female condoms have been evaluated from different perspectives, such as the qualitative approach [20–24], as well as the quantitative approach, which aims at a more standardized assessment of the construct. Among the latter, the Female Condom Attitudes Scale (FCAS [25]) has been the most widely used measure, and it has been validated using appropriate indicators.

The FCAS was developed by Neilands and Choi [25] with the purpose of providing researchers a standardized tool to evaluate attitudes toward the female condom given that this prophylactic method has become an alternative solution to prevent STIs and unplanned pregnancies [24]. Neilands and Choi [25] also conducted the validation of the short version of the scale, which includes 14 of the 30 original items distributed among five factors: *Sexual pleasure enhancement, Inconvenience, Improved prophylaxis, Sexual pleasure inhibition, and Insertion reluctance.* From the psychometric perspective, the short version was proven to be more robust than the extended version [25]. Answers are arranged on a four-point Likert-type scale *(Completely disagree, Partially disagree, Partially agree, and Completely agree)*. Nine of the items are inverted reversed.

Given the absence of Spanish adaptations for this scale (there is only a Portuguese adaptation by Gomes, Dos Santos, and Pernas [26] and the importance of accurately evaluating attitudes toward the female condom specifically, the purpose of the present study was to translate, adapt, and validate the FCAS for Spanish-speaking populations using a sample of young Colombian women and to explore its usage and knowledge about it in the country.

## Methods

Sampling was carried out using a non-probabilistic incidental approach which rendered a sample of 387 Colombian women aged between 18 and 42 years (*M* = 23.68, *SD* = 5.1). An 81% (*n* = 307) of the sample lived in the city of Bogotá, 3.2% (*n* = 12) in the city of Medellín, 2.4% (*n* = 9%) in the city of Barranquilla, and the remaining 13.4% (*n* = 51) lived in other 29 cities throughout the country. Inclusion criteria were that subjects were Colombian citizens living in the country, aged 18 or older, and had accepted written informed consent. Information on the sociodemographic and psychoaffective characteristics of the sample are shown in Table 1.

**Table 1:** Sociodemographic characteristics of the sample

		*M* (*SD*) or *n*(%)
Age		23.68 (5.1)
Approximate monthly salary	Equal or less than one minimum wage	203 (53%)
	Between 1 and 2 minimal wages	76 (20.1%)
	Between 2 and 3 minimal wages	45 (11.9%)
	Between 3 and 4 minimal wages	22 (5.8%)
	More than 4 minimum wages	16 (4.7%)
Sexual orientation		
	Asexual	5 (1%)
	Exclusively heterosexual	312 (82.3%)
	2	41 (10.8%)
	3	8 (2.1%)
	4	6 (1.6%)
	5	3 (0.8%)
	6	2 (0.5%)
	Exclusively homosexual	5 (1.3%)
Marital Status		
	Married	23 (61%)
	Single	304 (80.2%)
	Common law marriage	46 (12.1%)
	Separated	6 (1.6%)
Stable partner		
	Yes.	246 (64%)
	No	131 (34.6)
Attends religious services		
	Never	101 (26.6%)
	Once a year	149 (39.3)
	Once a month	64 (16.9%)
	At least once every three weeks	7 (1.8%)
	At least once every two weeks	8 (2.1%)
	At least once per week	46 (12.1%)
	Every day	3 (0.8%)

*Note.* Variables are shown as absolute values and percentages. Current legal minimum wage expressed in COP 737,717 (USD = 251.60). A total of 25 women decided to do not answer the salary question, the rest of missed answers are not superior to 10

### Sociodemographic questionnaire

Different sociodemographic characteristics of the participants were measured using an ad-hoc semi-structured survey that had been previously used in other studies focusing on the Colombian context [8]. Participants were asked about age, sex, educational level, religiousness, approximate monthly wage, marital status, sexual orientation, and whether they had a couple relationship. They were also asked if they had heard about, seen, touched, or used female condoms.

### Female condom attitude scale (FCAS [25])

In its short version, this instrument consists of 14 items, which are scored using a four-point Likert-type scale: 1 = *Strongly disagree*, 2 = *Partially disagree*, 3 = *Partially agree*, 4 = *Strongly agree*. The scale focuses on five factors: *Sexual pleasure enhancement, Inconvenience, Improved prophylaxis, Sexual pleasure inhibition, and Insertion reluctance.* Subscale internal consistency ranged from .68 to .87 (original version). The questionnaire is presented as Additional file 1.

### Sexual opinion survey (SOS [27])

The present study used the short version of this scale validated for Colombian population [28]. The SOS consists of six items and uses a seven-point Likert-type scale. Its single-factor structure evaluates respondents along a spectrum from *Erotophilia* (positive attitudes toward sexuality) and *Erotophobia* (negative attitudes toward sexuality). High scores in the scale reflect a higher degree of positive attitudes toward sexuality. The reliability of the Colombian version of the scale was α = .85; reliability in the present study was α = .81. A sample item is: “I find it exciting to think about engaging in sexual intercourse”.

### Sexual assertiveness scale (SAS [29])

The dimension *Sexually Transmitted Diseases-Unplanned Pregnancy* (STD-P) of the SAS version for Colombia [30] was used; this dimension has a reliability of α = .89. The subscale is composed of three items using a five-point Likert-type scale, whose response alternatives range from 0 = *Never* to 4 = *Always*. A sample item is: “When I have sex with my partner I make sure to use a condom or other latex barrier”. For the present study, alpha was equal to .88.

### UCLA multidimensional condom attitudes scale (MCAS [31]

A version adapted for Colombia was employed [32]. The scale focuses on five factors: Reliability and effectiveness of condoms, sexual Pleasure associated with condom use, Stigma attached to persons who use condoms, Embarrassment about negotiation and use of condoms, and Embarrassment about the purchase of condoms. It includes 25 items with seven Likert-type response alternatives. The reliability of the scale ranges from α = .74 to α = .94. Higher scores represent more probabilities of using condoms. A sample item is: “Using condoms consistently for vaginal, anal, or oral sex is a good way to prevent pregnancies and sexual infections”. Alphas ranging from .66 to .86 were observed in this version.

#### Procedure

The FCAS was translated according to recommendations [33, 34]. As a first step, authorization to carry out the validation of the short version of the FCAS were obtained from the authors of the original version. Secondly, scale translation was initiated; two certified translators worked from English into Spanish and two forward translations were thus obtained. In the next step, a group of sexology and psychometrics experts with good English skills revised content correspondence between the original version and the Colombian version with the purpose of preserving the conceptual and cultural equivalence between the original English items and the new Spanish ones as recommended [35]. Thus, any omitted/added information observed between the language versions was analyzed and reviewed.

The final version of the scale was administered online on the SurveyMonkey© platform. After conducting a pilot survey, the instrument was published on the Facebook© social network from 23 March 2017 until 11 April 2017. The survey could be responded to using a laptop, smartphone, or tablet device. Participants anonymity and results were maintained confidential at all times.

#### Analysis of data

Results were analyzed using R software [36]. A polychoric matrix was constructed according to recommendations by Gadermann, Guhn, and Zumbo [37] for both Exploratory Factor Analysis (EFA) and for obtaining ordinal alpha and item properties. Tools such as ggplot2 [38], Psych [39] and corrplot [40] were also used. Raw data can be consulted in: 10.6084/m9.figshare.9876299

## Results

In a first step, the polychoric matrix was analyzed by EFA using Maximum Likelihood (ml) as extraction method, as well as Varimax rotation. Parallel Analysis (PA) was also conducted to estimate the number of factors making up the dimensionality. PA revealed the existence of four factors, as opposed to the five factors put forward by the original version. Nevertheless, items 1, 2, 3, 7, and 8 grouped themselves around a common factor; all of these items indicate a latent comparison between the male and female condom, except item 1, which does so more subtly. This factor could be coherently referred to a linguistic issue; however, from a theoretical point of view (related with attitudes) its significance is low, so the decision was made to repeat EFA with a fifth factor (using Kaiser criterion) in order to replicate the factor structure of the original version and to align our work with the theoretical proposal of the scale’s authors. Table 2 shows the weights of the items and the variance of each factor.

**Table 2:** Exploratory factor analysis (EFA) and Cronbach’s alpha for the short version of the Female Condom Attitude Scale (FCAS)

Items	Sexual pleasure enhancement^a^	Inconvenience	Improved prophylaxis	Sexual pleasure inhibition	Insertion reluctance
FCAS1	.66				
FCAS2	.99				
FCAS3^a^	.53		.35		
FCAS4		.63			
FCAS5		.80			
FCAS6		.80			
FCAS7			.77		
FCAS8			.80		
FCAS9				.67	
FCAS10				.82	
FCAS11				.77	
FCAS12				.70	
FCAS13					.94
FCAS14					.84
% of explained variance	12%^a^	14%	11%	18%	13%

Note. ^a^Item 3 was eliminated from version fitting. n for this analysis = 379

In general, factorization reflected the original version, except for item 3 “the female condom is better than the male condom”, which saturated for two factors, although neither of them was the inconvenience factor (where, theoretically, it should occur). Additionally, this item saturates in two factors, and we advance that it degrades the psychometric properties of the scale, so from now on it will be eliminated; the elimination of this item degrades the factor’s variance only by 1%.

Table 3 presents some of the items’ psychometric properties. These results were also calculated using the polychoric matrix, therefore, the corrected item-total correlations are polychoric and obtained alpha is the ordinal.

**Table 3:** Psychometric measures of Female Condom Attitude Scale

Item	*M*	*SD*	r^*c*^_*i-t*_	α-factor	α-item	*M* (*SD*)
FCAS1	2.49	0.88	.68	.81	–	4.90 (1.53)
FCAS2	2.40	0.83	.68		–	
FCAS4	2.29	1.02	.64	.84	.84	
FCAS5	2.58	0.88	.74		.75	7.25 (2.40)
FCAS6	2.37	0.95	.74		.75	
FCAS7	2.4	0.90	.68	.81	–	
FCAS8	2.49	0.97	.68		–	4.90 (1.68)
FCAS9	2.67	0.81	.67	.86	.85	
FCAS10	2.77	0.84	.77		.80	
FCAS11	2.96	0.78	.73		.82	11.33 (2.67)
FCAS12	2.96	0.89	.69		.84	
FCAS13	2.98	1.01	.91	.96	–	5.94 (1.95)
FCAS14	2.99	1.02	.91		–	

*M* Mean, *SD* Standard deviation, *r*^*c*^_*i-t*_ corrected total correlation of elements, *α-factor* Ordinal alpha by factors, *α-item* Ordinal alpha if element is eliminated. n for this analysis = 379

**Fig. 1:**
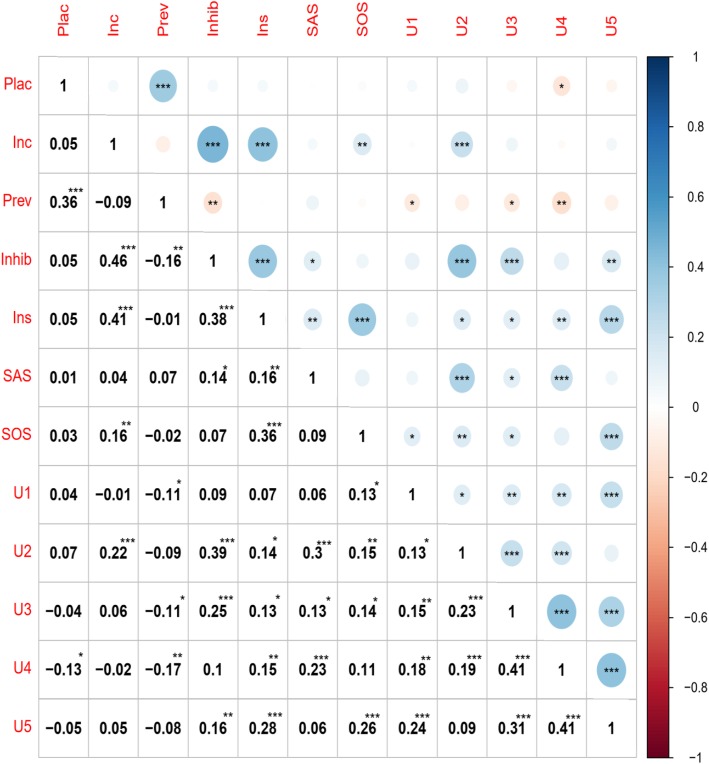
Correlation matrix for the variables used in the present study. * = *p* > .05, ** = *p* > .01, *** = *p* > .001. The reader is reminded that higher FCAS scores indicate more positive attitudes. Plea = FCAS (Sexual pleasure enhancement); Inc. = FCAS (Inconveniencia); Prev = FCAS (Improved prophylaxis); Inhib = FCAS (Sexual pleasure inhibition); Ins = FCAS (Insertion reluctance); SAS = Sexual Assertiveness Use and Negotiation of Condom; SOS = General Attitudes Toward Sexuality; U1 = Reliability and Effectiveness of Male Condom; U2 = Pleasure associated with male condom; U3 = Stigma associated with male condom; U4 = Embarrassment about negotiation and use of male condom; U5 = Embarrassment about purchasing male condom

Figure 1 shows the criterion validity for the five factors observed in the scale used to measure attitudes toward female condom as well as in other scales and subscales used as criterion. In general, observed relationships among the five factors of the FCAS were from moderate to nil, while the correlations between the factors and the criterion variables were from low to moderate.

**Fig. 2:**
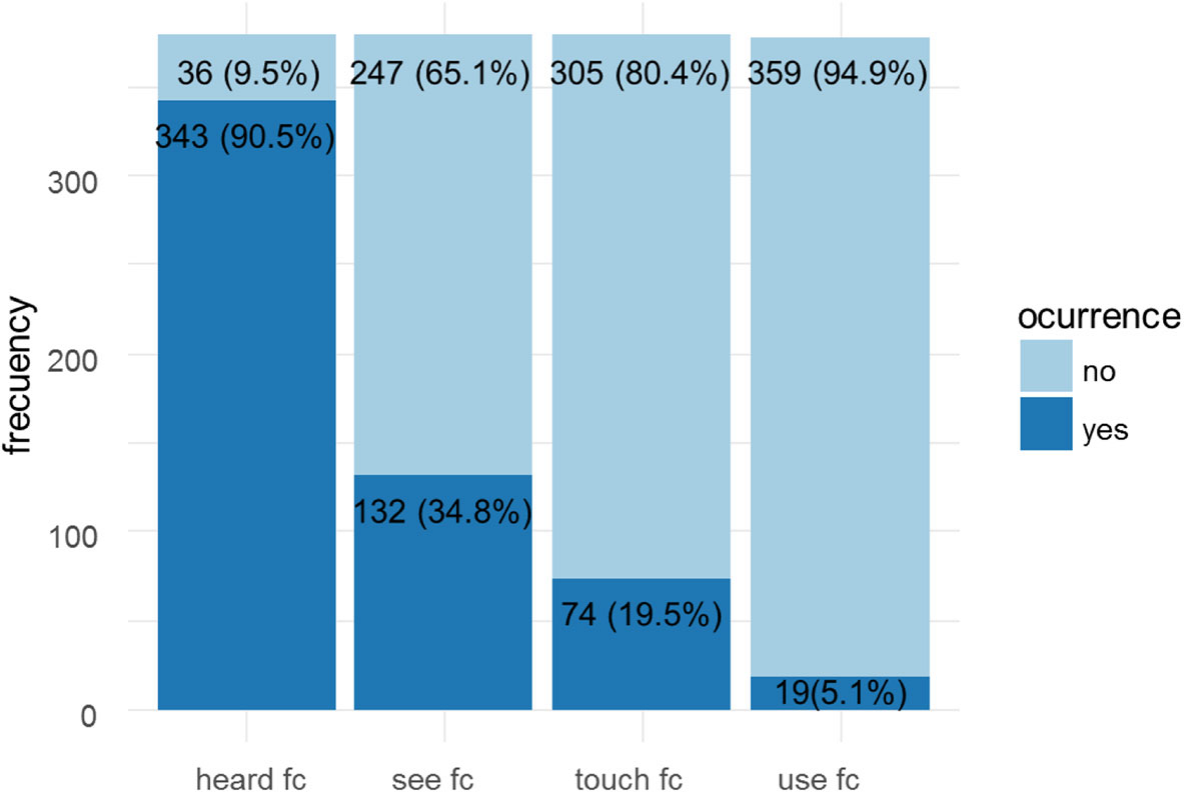
Frequency of interaction with female condom among women (up to 9 women did not answered this questions)

Finally, as an extension of the validity of the scale, we sought to explore variables related to knowledge and use of female condom among young Colombian women. Figure 2 shows the frequency with which women had heard about the existence of female condoms, had seen one, had touched one, and whether they had actually used it for sexual intercourse. In addition, no significant differences were observed between having heard about FC, having seen a FC, having touched a FC, or having used a FC with the attitudes toward the FC except for FC use in the Pleasure subscale t (374) = 2.19; *p* = 0.03; *d* = .50 (FC use *M* = 4.15(*SD* = 1.64) and not using FC *M* = 4.94(*SD* = 1.51)).

## Discussion

To the best of our knowledge, there are currently no published studies focused on the female condom in Colombia, and in fact, the lack of instruments evaluating psychological variables associated with the device extends to the rest of the Spanish-speaking world. As a contribution to fill this gap, the objectives of the present study were to translate and adapt the short version of the *Female Condom Attitude Scale* (FCAS [25]) for Spanish language and to validate the scale using a Colombian sample of women. For those purposes, the content of the scale was translated to Colombian Spanish, reliability indices were obtained, EFA was carried out, and the external validity of the scale with respect to other measures (SAS, SOS, and MCAS) could be confirmed. In general, as with the version by Neilands & Choi [25], the scale was composed of five factors (sexual pleasure enhancement, inconvenience, improved prophylaxis, sexual pleasure inhibition, insertion reluctance). Correlations between the FCAS and the other measures were as expected based on the theory [15], were attitudes will be a good predictor of condom use related variables.

There were no difficulties to translate and adapt the scale into Spanish. Also, as with the original short version [25], five factors were observed, although this is different from results obtained in Portugal [26]. In our version, item 3 had to be eliminated due to its saturation in two factors, both different from the factor predicted by the theory [25]; moreover, the item degraded the metric properties of the scale. The percentage of explained variance is slightly higher in our version than in the Portuguese version [26]. Psychometric properties were appropriate for our version. The factors’ Cronbach’s alphas ranged between .81 and .96, which indicates internal consistency and reliability; these two characteristics were also higher than those obtained in previous versions of the FCAS [25, 26].

The relationships between the factors of the scale are consistent with the theory and present acceptable criterion validity. Inconvenience and sexual inhibition were related in a positive and moderate way, as it was reported for the original version [25]. Insertion reluctance was expected to correlate with sexual pleasure inhibition. There is a general interrelationship between the FCAS subscales. Concerning relationships between FCAS and other measures (i.e. discriminant validity), they were found to be low or moderate in most cases. Higgins and Wang [41] observed associations between pleasure-related attitudes and the use of male condom, which is similar to the association between condom insertion reluctance, inconvenience, and attitudes toward sexuality (i.e. SOS). Despite that the constructs proposed by Higgins and Wang [41] are slightly different from the constructs targeted by the FCAS and the SOS (i.e., attitudes toward sexuality), there is a possible relationship between pleasure-related sexual attitudes and attitudes toward sexuality in general, an association that would support our observations. Nevertheless, this association was not observed for male condoms [42]. Relationships observed between SAS (i.e. sexual assertiveness) and FCAS were as expected; sexual assertiveness has been shown to be a predictor of the use of contraceptive intravaginal devices [43], and is associated with difficulties to obtain sexual pleasure [44, 45]. Finally, attitudes toward the female condom were compared with those toward the male condom. In this case, different attitudes toward male condom are consistently related to each other, but they are completely independent of attitudes toward female condom. This points out the necessity of evaluating specific attitudes toward the male and toward the female device separately when required since they seem to be two different constructs.

The outlook for the availability of female condoms around the world is unpromising. Consistent with the results of the present study, focused on Colombia—a low- and middle-income economy—, a vast majority of women in developing and developed countries (i.e., at least 90% of women) have heard about this type of condom, but there are noticeable differences when it comes to seeing or using the device [24]. Less than 25% of women in the United States have used the female condom [24], and the use of intravaginal contraceptive methods in Africa and Latin America is below 5%, which reflects the observations reported in the present paper, [46]. Although the specific proportions vary depending on the country, the use of female condoms tends to be higher in developed countries, and the use of male condoms is consistently more widespread [24, 47, 48]. There are different reasons why women would prefer not to use a female condom, some of them associated with the availability provided by the market [46, 49–51]. In any case, these limitations increase the risk associated with sexual behavior, and consequently, the spread of STDs and the number of unplanned pregnancies. Thus, easier access to female condoms could have a positive effect on people’s attitudes toward the device, which would in turn support prevention efforts based on its use. This scale can be an essential tool to assess attitudes toward female condoms before and after interventions focused on sexual health targeted at people who have never used it. It can also be used for evaluating effectiveness of programs based on health promotion and behavioral risk reduction theories [52] that include a female condom component, due to it can increase condom use. Finally, female condom is known in Colombia but seldom used in Colombia. Sexual health promotion programs in Colombia, should include female condom as one of the potential methods [53].

## Conclusion

Certain considerations should be taken into account before extrapolating the results of the present study. Although the psychometric properties of the adapted version meet the required reliability and validity standards [54], its results should be viewed with caution due to the lack of confirmatory factor analysis. It was not possible to evaluate the convergent validity of the FCAS against other Spanish-adapted and validated scales for measuring attitudes toward female condoms because FCAS is the first scale of its type available in Spanish. Neilands & Choi’s five-factor composition [25] and Gomes et al.’s three-factor composition [26] are two possible factor compositions for the scale; their adequacy for Colombians should be evaluated in the context of further research on attitudes toward the use of female condoms. In addition, the convergent validity of the instrument should be tested using a different instrument measuring the same construct. Finally, the properties of the scale must be evaluated in populations that could have contrasting attitudes toward the female condom, such as female sex workers and women who have been infected by HIV or other STIs. The empowerment that a female condom can provide to women might help to improve their attitudes toward the device [55, 56].

## Supplementary information

**Additional file 1.** Spanish version of the Female Condom Attitude Scale. In this section the Spanish version of the scale can be consulted.

## Data Availability

The datasets generated and/or analyzed during the current study are available in the Figshare repository: 10.6084/m9.figshare.9876299

## Abbreviations

AIDS: Acquired Immunodeficiency Syndrome
EFA: Exploratory Factor Analysis
FCAS: Female Condom Attitudes Scale
HIV: Human Immunodeficiency Virus
Inc.: FCAS (Inconvenience)
Inhib: FCAS (Sexual pleasure inhibition)
Ins: FCAS (Insertion reluctance)
M: Mean
MCAS: Multidimensional Condom Attitudes Scale
ML: Maximum Likelihood
PA: Parallel analysis
Plea: FCAS (Sexual pleasure enhancement)
Prev: FCAS (Improved prophylaxis)
rci-t: corrected total correlation of elements
SAS: Sexual Assertiveness Scale
SD: Standard Deviation
SOS: Sexual Opinion Survey
STD-P: Sexually Transmitted Diseases-Unplanned Pregnancy
STIs: Sexually Transmitted Infections
U1: Reliability and Effectiveness of Male Condom
U2: Pleasure associated with male condom
U3: Stigma associated with male condom
U4: Embarrassment about negotiation and use of male condom
U5: Embarrassment about purchasing male condom
UCLA: Univeristy California Los Angeles
α-factor: Ordinal alpha by factors;
α-item: Ordinal alpha if element is eliminated.
