# Supplementary material for: Spanish validation of female condom attitude scale and female condom use in Colombian young women
Source: BMC Womens Health. 2019 Oct 28;19:128. doi: 10.1186/s12905-019-0825-z (PMC6819378; doi:10.1186/s12905-019-0825-z)
Supplement: Supplementary file 1 — Additional file 1. Spanish version of the Female Condom Attitude Scale. In this section the Spanish version of the scale can be consulted. [file 12905_2019_825_MOESM1_ESM.docx]

*Female Condom Attitude Scale (Escala de Actitudes al Condón Femenino; FCAS; Neilands y Choi, 2002)* – *and Colombian validated version.*

| Muy en desacuerdo/ Disagree a lot | Un poco en desacuerdo/ Kind of disagree | Un poco de acuerdo/ Kind of agree | Muy de acuerdo/ ”Agree a lot |  |
| --- | --- | --- | --- | --- |
| 1. El condón femenino hace que el sexo sea mejor para las mujeres/ *Female condoms make sex better for women.* | 1 | 2 | 3 | 4 |
| 2. El condón femenino se siente más natural que el condón masculino tradicional/ *Female condoms feel more natural than regular male condoms.* | 1 | 2 | 3 | 4 |
| 3. El condón femenino es raro/ *Female condoms are weird.* | 1 | 2 | 3 | 4 |
| 4. El condón femenino es incómodo/*Female condoms are inconvenient.* | 1 | 2 | 3 | 4 |
| 5. El condón femenino es complicado/ *Female condoms are messy.* | 1 | 2 | 3 | 4 |
| 6. El condón femenino ofrece una mejor protección contra el embarazo no deseado que el condón masculino/ *Female condoms offer better protection against unwanted pregnancy than male condoms do.* | 1 | 2 | 3 | 4 |
| 7. El condón femenino ofrece una mejor protección contra las infecciones de transmisión sexual que el condón masculino. *Female condoms offer better protection against sexually transmitted diseases than male condoms do.* | 1 | 2 | 3 | 4 |
| 8. El sexo no se siente tan bien cuando se utiliza un condón femenino*/ Sex doesn’t feel as good when you use a female condom.* | 1 | 2 | 3 | 4 |
| 9. El condón femenino hace que sea difícil para una mujer tener un orgasmo (venirse). */ Female condoms make it hard for a woman to have an orgasm (cum).* | 1 | 2 | 3 | 4 |
| 10. El condón femenino hace que sea difícil para un hombre tener un orgasmo (venirse)/ *Female condoms make it hard for a man to have an orgasm (cum).* | 1 | 2 | 3 | 4 |
| 11. El condón femenino le quita la diversión al sexo/ *Female condoms take all the fun out of sex.* | 1 | 2 | 3 | 4 |
| 12. No te gusta tener que tocarte para introducirte el condón femenino/ *You don’t like having to touch yourself to put the female condom in.* | 1 | 2 | 3 | 4 |
| 13. No te gusta tener que usar tu dedo para introducirte el condón femenino/ *You don’t like having to use your finger to push the female condom in.* | 1 | 2 | 3 | 4 |

Note. From item 3 the numbering of this version does not coincide with the original. Thus item 3 is the number 4 of the original version and so on. English version showed here has to be cited as: Neilands T, Choi K. A validation and reduced form of the female condom attitudes scale. AIDS Educ Prev. 2002; doi: 10.1521/aeap.14.2.158.23903
